# The 2nd International Conference on Agricultural and Biological Sciences (ABS 2016): Shanghai, China. 23-26 July 2016

**DOI:** 10.1186/s12870-016-0904-3

**Published:** 2016-10-24

**Authors:** Lei Pei, Zhenhua Wang, Jinzhu Zhang, Wenhao Li, Adeline S. Y. Ting, Yiing Y. Chow, Sadequr Rahman, Rongchang Wei, Zhang Hu, Sidong Li, Chuyan Li, Zijuan Li, Guoxia Wang, Yuzhen Yang, Gang Chen, Qing Luo, Guoxia Wang, Ruixia Liu, Yuzhen Yang, Lipei Chen, Zhiyang Lie, Tongtong Zhou, Weilong Huang, Li Xue, Jie Li, Zhuomin Wang, Li Xue, Ismail Bezirganoglu, Pınar Uysal, Shuihua Wang, Zhihai Lu, Jiquan Yang, Yudong Zhang, John Liu, Ling Wei, Shufang Chen, Preetha Phillips, Zhengchao Dong, Hong Li, Xutong Wang, Fengliang Zhao, Guisheng Yang, Haijie Huang, Li Zhao, Weijian Huang, Jinhui Wang, Zhongrun Zhang, Xiaojuan Li, Ning Xu, Guofu Zhou, Ming Wan, Qi Lin, Fanyun Meng, Jianxiu Li, Yichang Chen, Koayung Yu, Chunpin Chang, Zijuan Zhou, Peixi Su, Rui Shi, Tingting Xie, Rui Shi, Peixi Su, Zijuan Zhou, Xuelan Liu, Yan Zhang, Xiangfa Wei, Chong Wu, Yanlei Yin, Lijuan Feng, Xuemei Yang, Fei Wang, Hua Wang, Huifa Zhuang, Zihui Zhu, Hui Wang

**Affiliations:** 1 College of Water & Architectural Engineering, Shihezi University, Shihezi City, 832000 Xinjiang China; 2 Key Laboratory of Modern Water-Saving Irrigation Corp, Shihezi University, Shihezi City, 832000 Xinjiang China; 3 School of Science, Monash University Malaysia, Jalan Lagoon Selatan, 46150 Bandar Sunway, Selangor Malaysia; 4 Guangxi Botanical Garden of Medicinal Plants, Nanning, Guangxi Province 530023 China; 5 Department of Chemistry, College of Science, Guangdong Ocean University, Zhanjiang, 524088 China; 6 School of Life Science, Zhengzhou Normal University, Zhengzhou, 450044 China; 7 School of Life Science, Zhengzhou Normal University, Zhengzhou, 450044 China; 8 College of Forestry and Landscape Architecture, South China Agricultural University, Guangzhou, 510642 China; 9 College of Forestry and Landscape Architecture, South China Agricultural University, Guangzhou, 510642 China; 10 Deparment of Molecular Biology and Genetic, Erzurum Technical University, Erzurum, 442 Turkey; 11 Ministry of Food, Agriculture and Husbandry, Eastern Anatolia Agriculture Research Institute, Erzurum, 442 Turkey; 12 Nanjing Normal University, Nanjing, Jiangsu 210023 China; 13 Key laboratory of symbolic computation and knowledge engineering of ministry of education, Jilin University, Changchun, Jilin 130012 China; 14 Michigan State University, East Lansing, MI 48823 USA; 15 Shanghai Jiaotong University, Shanghai, 200030 China; 16 University of Electronic Science and Technology of China, Chengdu, Sichuan 611731 China; 17 Shepherd University, Shepherdstown, WV 25443 USA; 18 West Virginia University, Morgantown, WV 26505 USA; 19 New York State Psychiatric Institute, New York, NY 10032 USA; 20 Environment and Plant Protection Institute, Chinese Academy of Tropical Agricultural Science, Haikou, Hainan 571101 China; 21 Tropical Crops Genetic Resources Institute, CATAS, Danzhou, 571737 China; 22 College of Applied Science and Technology, Hainan University, Danzhou, 571737 China; 23 Beijing Area Major Laboratory of Protection and Utilization of Traditional Chinese Medicine, Beijing Normal University, Beijing, 100875 China; 24 State Key Laboratory Breeding Base of Dao-di Herbs, National Resource Center for Chinese Materia Medica, China Academy of Chinese Medical Sciences, Beijing, 100700 China; 25 Shandong University of Traditional Chinense Medicine, Jinan, 250014 China; 26 Shandong Hongjitang Museum, Jinan, 250103 China; 27 Institute of Botany, the Chinese Academy of Sciences, Beijing, 100093 China; 28 National Chung Hsing University, Taichung, Taiwan 402 Taiwan; 29 Ling Tung University, Taichung, Taiwan 408 Taiwan; 30 Chung Chou University of Science and Technology, Changhua, Taiwan 510 Taiwan; 31 Key Laboratory of Land Surface Process and Climate Change in Cold and Arid Regions, Cold and Arid Regions Environmental and Engineering Research Institute, Chinese Academy of Sciences, Lanzhou, 730000 China; 32 Key Laboratory of Land Surface Process and Climate Change in Cold and Arid Regions, Cold and Arid Regions Environmental and Engineering Research Institute, Chinese Academy of Sciences, Lanzhou, 730000 China; 33 Poultry Research Institute, Shandong Academy of Agricultural Sciences, Jinan, 250023 China; 34 Shandong Institute of Pomology, Taian, 271000 Shandong China; 35 Institute of Spice and Beverage Research, CATAS, Wanning, 571533 China; 36 Tropical Crops Genetic Resources Institute, CATAS, Danzhou, 571737 China

## Abstract

01 The influence of soil salt content on the photosynthetic characteristics of spring wheat with trickle irrigation

Lei Pei, Zhenhua Wang, Jinzhu Zhang, Wenhao Li

02 Comparing growth of fast-growing and slow-growing endophytes in plants via ergosterol quantification

Adeline SY Ting, Yiing Y Chow, Sadequr Rahman

03 Transcriptome and digital gene expression analysis identifies putative triterpene saponin-biosynthetic genes of *Panax notoginseng*

Rongchang Wei

04 Chitosan-assisted isolation and antioxidant evaluation of flavonoids from *Sophora japonica* L.

Zhang Hu, Sidong Li, Chuyan Li, Zijuan Li

05 Two kinds of new characteristics of the ektexine ornamentation of ginkgo pollen

Guoxia Wang, Yuzhen Yang, Gang Chen, Qing Luo

06 Analysis of nutrient and medicinal ingredients of Ginkgo pollen in different regions

Guoxia Wang, Ruixia Liu, Yuzhen Yang, Lipei Chen

07 Photosynthetic performance of greening seedlings of seven species to drought stress

Zhiyang Lie, Tongtong Zhou, Weilong Huang, Li Xue

08 Changes of fluorescence parameters of greening seedlings of seven species under drought stress

Jie Li, Zhuomin Wang, Li Xue

09 Mammalian sex hormone affects regeneration capacity and enzymes activity of Triticale L in vitro culture

Ismail Bezirganoglu, Pınar Uysal

10 Fractional Fourier entropy increases the recognition rate of fruit type detection

Shuihua Wang, Zhihai Lu, Jiquan Yang, Yudong Zhang, John Liu, Ling Wei, Shufang Chen, Preetha Phillips, Zhengchao Dong

11 Banana-peanut intercropping reduces *Fusarium* wilt disease in banana from enhancing soil bacterial microorganisms and leaf nutrition

Hong Li, Xutong Wang, Fengliang Zhao, Guisheng Yang

12 Manganese stress impairs stem ureide nitrogen fixation in yardlong-bean plants in the acidic environment

Hong Li

13 A new pest control method for *Rhytidodera bowringii* Larvae

Haijie Huang, Li Zhao, Weijian Huang, Jinhui Wang, Zhongrun Zhang

14 Research on the seed-like Fruits of Subg. Sclarea of *Salvia* of Labiatae in China

Xiaojuan Li, Ning Xu, Guofu Zhou, Ming Wan, Qi Lin, Fanyun Meng, Jianxiu Li

15 Three pulling resistance models of pioneer plant in landslide area

Yichang Chen, Koayung Yu, Chunpin Chang

16 The comparison of physiological and biochemical mechanisms of *Reaumuria soongorica* and *Salsola passerine* in different growth pattern

Zijuan Zhou, Peixi Su, Rui Shi, Tingting Xie

17 Resources use efficiency of the cosmopolitan plant *Potentilla anserina* L. in different alpine habitats in China

Rui Shi, Peixi Su, Zijuan Zhou

18 Cloning of PPDK gene from *Red Amaran*and transformation of *Alfalfa*

Xuelan Liu, Yan Zhang, Xiangfa Wei

19 Variation and cluster analysis of morphological characters and nutrient content of *Chucrasia tabularis* seed

Chong Wu, Yanlei Yin, Lijuan Feng, Xuemei Yang, Fei Wang

20 Effect of the planting density of the areca nut on the growth of intercropped Vanilla

Hua Wang, Huifa Zhuang, Zihui Zhu, Hui Wang

## 01 The influence of soil salt content on the photosynthetic characteristics of spring wheat with trickle irrigation

### Lei Pei^1,2^, Zhenhua Wang^1,2^, Jinzhu Zhang^1,2^, Wenhao Li^1,2^

#### ^1^College of Water & Architectural Engineering, Shihezi University, Shihezi City 832000, Xinjiang, China; ^2^Key Laboratory of Modern Water-Saving Irrigation Corp, Shihezi University, Shihezi City 832000, Xinjiang, China

##### **Correspondence:** Zhenhua Wang (wzh2002027@163.com); Jinzhu Zhang (xjshzzjz@sina.cn)

To understand the influence of salt content in soil on the photosynthetic physiological indexes of spring wheat with trickle irrigation, the changes of the photosynthetic physiological indexes of spring wheat and the stomatal limitation and non-stomatal limitation features of photosynthesis were analyzed so as to provide theoretical basis and technical support for spring wheat cultivation in saline-alkali soils with trickle irrigation. Pot experiment was conducted to investigate changes of the photosynthetic characteristics of spring wheat under different salt contents in soil (0.15 % (CK), 0.80 %, 1.70 %, and 2.60 %). The results showed that under different conditions of salt content, the daily variations of photosynthetic physiological indexes were similar, and the “noon nap” phenomenon during photosynthesis was obvious. Leaf stomatal conductance of spring wheat was suppressed by the salts in soil, resulting in the decreases of net photosynthetic rate, water utilization efficiency and solar energy utilization efficiency; leaf photosynthesis of spring wheat was restrained by the simultaneous existing of stomatal and non-stomatal factors; decrease of the net photosynthetic rate was mainly caused by stomatal factors under low salt content conditions and by non-stomatal factors under high salt content conditions. The spring wheat leaves had relatively high net photosynthetic rates in soils with initial salt contents of 0.15 % and 0.80 %. According to the results from this study, it is preliminarily concluded that spring wheat with trickle irrigation can be planted in saline-alkali soils with salt content lower than mild salinization soil.

**Keywords:** Trickle irrigation; Spring wheat; Salt content in soil; Photosynthetic characteristics; Stomatal and non-stomatal factor

## 02 Comparing growth of fast-growing and slow-growing endophytes in plants via ergosterol quantification

### Adeline SY Ting, Yiing Y Chow, Sadequr Rahman

#### School of Science, Monash University Malaysia, Jalan Lagoon Selatan, 46150 Bandar Sunway, Selangor, Malaysia

##### **Correspondence:** Adeline SY Ting (adeline.ting@monash.edu)

The ability of endophytes to grow in plant tissues is poorly understood as the plant-endophyte association is a complex interaction. It has been hypothesized that poor endophyte growth in plant tissues may consequently result in inferior colonization and expression of antifungal activities. It is pertinent that the growth of the various endophytes (fast- and slow-growing endophytes) are studied in planta, particularly when fast- and slow-growing endophytes competitively exclude pathogens and produce strong antifungal compounds, respectively. In this study, plantlets were first inoculated with fast-growing endophytes (*Trichoderma asperellum* T2, *Diaporthe phaseolorum* WAA02) and slow-growing endophytes (*Penicillium citrinum* BTF08, *Ganoderma boninense* or Gb). Their growth in planta was determined based on ergosterol extraction (microwave-assisted extraction method) and quantified with HPLC. Results revealed that slow-growing endophyte BTF08 is naturally richer in ergosterol (66.4 μg per 2 g biomass) compared to fast-growing endophytes (WAA02 and T2 with 13.4 and 39.3 μg per 2 g biomass, respectively). Unlike BTF08, Gb (22.6 μg per 2 g biomass) has lesser ergosterol, suggesting that sporulation may be another factor contributing to ergosterol content. In plants, fast-growing endophytes (WAA02, T2) have the edge of growing better and succeeding in colonization and establishment with 0.292 and 0.055 g of fungal biomass per g of plant tissue estimated, respectively. Their biomass was superior to biomass of slow-growing endophytes (BTF08 and Gb) with only 0.045 and 0.026 g of fungal biomass detected per g of plant tissue, respectively. The influence of endophyte growth and subsequent disease suppression will be evaluated in future.

## 03 Transcriptome and digital gene expression analysis identifies putative triterpene saponin-biosynthetic genes of *Panax notoginseng*

### Rongchang Wei (wrc830612@163.com)

#### Guangxi Botanical Garden of Medicinal Plants, Nanning, Guangxi Province 530023, People’s Republic of China

**Background:** *Panax notoginseng* is a well-known Chinese medicinal herb in the world. The principal bioactive constituents of *P. notoginseng* are triterpene saponins, including ginsenoside Rg_1_, ginsenoside Rb_1_ and notoginsenosides R_1_. However, little is known about the biosynthetic pathways of Rg_1_, Rb_1_ and R_1_.

**Result:** Equal amount of RNA obtained at four stages of root development in which triterpene saponins levels change were pooled and sequenced using Illumina/Solexa platform. A total of 55,155,156 clean reads were generated and assembled into 96,704 unigenes. Based on the Kyoto Encyclopedia of Genes and Genomes, 907 genes related to triterpene saponins backbone biosynthesis were discovered. Digital gene expression (DGE) profiles of four root developmental stages were performed using Solexa sequencing. According to triterpene saponins levels and genes expression profiles, eight *UDP-glucosyltransferases* and nine *cytochrome P450s* were discovered to be involved in triterpene saponins biosynthesis of *P. notoginseng*.

**Conclusion:** This study identified a large number of candidate genes related to triterpene saponins backbone biosynthesis of *P. notoginseng*, including eight *UDP-glucosyltransferases* and nine *cytochrome P450s*. It will provide invaluable resources for understanding the biosynthetic pathways of Rg_1_, Rb_1_ and R_1_.

**Keywords:** *Panax notoginseng*, Triterpene saponins biosynthesis, Transcriptome, DGE

**Acknowledgements**

This study was supported by the Guangxi Natural Science Foundation Program of China (2014GXNSFAA118254 and 2013GXNSFAA019207).

## 04 Chitosan-assisted isolation and antioxidant evaluation of flavonoids from *Sophora japonica* L.

### Zhang Hu, Sidong Li, Chuyan Li, Zijuan Li

#### Department of Chemistry, College of Science, Guangdong Ocean University, Zhanjiang 524088, China

##### **Correspondence:** Zhang Hu (huzhangqyx@126.com)

*Sophora japonica* L. has been used as hemostatic agent in diseases caused by abnormal hemostatic mechanisms in traditional Chinese medicine since ancient time. The total flavonoids were extracted from *Sophora japonica* L. by ultrasonic technique and isolated via chitosan-assisted polyamide method. On the base of single factor experiments, Box-Behnken experimental design was adopted to optimize the process conditions including ultrasonic temperature, ratio of liquid to raw material, processing time and amount of chitosan. By response surface methodology analysis, the optimal process conditions were obtained as follows: ultrasonic temperature 60 °C, ratio of liquid to raw material 22:1(v/w), processing time 46 min and amount of chitosan 8‰ (w/v). The experimental results showed that the yield of the total flavonoids was 19.54 % and the purity was 46.27 %. Further determination of antioxidant activity of the total flavonoids was also studied.The results showed that the achieved flavonoids exhibited potent hydroxyl and superoxide anion free radicals scavenging capacity in a concentration-dependent manner. In conclusion, a novel and efficient extraction and chitosan-assisted isolation technique for flavonoids from *Sophora japonica* L. was developed and the obtained flavonoids were of significantly antioxidant activity.

**Acknowledgements**

We gratefully acknowledge the financial support by Natural Science Foundation of Guangdong Province (2016A030308009), Scientific and Technological Planning Project of Guangdong Province (2015A020216019), and Project of Enhancing School with Innovation of Guangdong Ocean University (2015KTSCX053, 2014KZDXM038 and GDOU2015050253).

## 05 Two kinds of new characteristics of the ektexine ornamentation of ginkgo pollen

### Guoxia Wang, Yuzhen Yang, Gang Chen, Qing Luo

#### School of Life Science, Zhengzhou Normal University, Zhengzhou 450044, P. R. China

##### **Correspondence:** Guoxia Wang (wgxia191919@sina.com.cn)

The morphological characteristics of pollen is an important reference for the classification of plants, the relationship between species and the classification of varieties. China is the origin of *Ginkgo biloba*. Ancient trees are the best carrier of genetic information. The pollens of 13 ancient male ginkgo trees in Henan Province were observed through scanning electron microscopy. The results showed that the ginkgo pollen had approximately similar outside morphology, and they were all olive shaped and had a bourgeon channels which was about the length of pollen. The pollen length ranged from 30.05 μm to 35.63 μm, the width of pollen ranged from 13.93 μm to 17.73 μm, and the ratio of the pole length to the equator(P/E) ranged from 1.77 to 2.45. In the description of the characteristics of ginkgo pollen ektexine ornamentation, only stripe ornamentation, punctate ornamentation and ridge protuberance were used in the past. The study not only found the above ornamentation features, but also found the scale shaped ridge protuberance and conical protuberance which were previously not reported, especially the conical protuberance of Songxian3 was larger and its base length more than 1 μm. The morphology of ginkgo pollen was complex and diverse.

**Acknowledgements**

Supported by a project grant from the Science and technology research key project of Henan Province Office of Education (13A180349).

## 06 Analysis of nutrient and medicinal ingredients of Ginkgo pollen in different regions

### Guoxia Wang, Ruixia Liu, Yuzhen Yang, Lipei Chen

#### School of Life Science, Zhengzhou Normal University, Zhengzhou 450044, P. R. China

##### **Correspondence:** Guoxia Wang (wgxia191919@sina.com.cn)

Ginkgo pollens are rich in essential amino acids, vitamins, flavonoids and other ingredients, can be widely used in the development of health products and other nutrition utilization, but whether there are differences between Ginkgo pollens from different regions had not been finalized. The contents of vitamin B1, vitamin B2, vitamin C, amino acids, total flavonoids of Ginkgo pollen were measured from 18 Chinese Cities. The average contents of vitamin B1, vitamin B2, vitamin C, amino acids, total flavonoids of Ginkgo pollens of 18 cities were respectively 0.1211 mg.g^−1^, 0.6476 mg.g^−1^, 0.1135 mg.g^−1^, 225.2 mg.g^−1^ and 27.41 mg.g^−1^. The highest three of vitamin B1 were Gunlin, Lueyang and Pizhou amount the 18 regions, respectively was 0.2239 mg.g^−1^, 0.2167 mg.g^−1^ and 0.1912 mg.g^−1^; the highest three of vitamin B2 were Pizhou, Fenghua and Doujiangyan, respectively was 1.1875 mg.g^−1^, 1.0442 mg.g^−1^and 0.9333 mg.g^−1^; the highest three of vitamin C were Xixia, Fenghua and Tengchong, respectively was 0.2061 mg.g^−1^, 0.1882 mg.g^−1^ and 0.1828 mg.g^−1^; the highest three of amino acids were Guilin, Changxing and Xinxian, respectively was 253.3 mg.g^−1^, 252.8 mg.g^−1^ and 249.8 mg.g^−1^; the highest three of total flavonoids were Nanxiong, Kangxian and Lueyang, respectively was 36.58 mg.g^−1^, 32.93 mg.g^−1^ and 31.80 mg.g^−1^. The result showed that there were significant differences in the nutrient and medicinal ingredients from diffrent regions.

**Acknowledgements**

Supported by a project grant from the science and technology research key project of Henan Province Office of Education (13A180349).

## 07 Photosynthetic performance of greening seedlings of seven species to drought stress

### Zhiyang Lie, Tongtong Zhou, Weilong Huang, Li Xue

#### College of Forestry and Landscape Architecture, South China Agricultural University, Guangzhou 510642, China

##### **Correspondence:** Li Xue (forxue@scau.edu.cn)

Studies were conducted on photosynthetic characteristics of seedlings of *Syzygium hainanense, Phoebe sheareri, Radermachera sinica, Ilex rotunda, Ficus microcarpa, Carallia diplopetela* and *Artocarpus nitidus* ssp. *lingnanensis* under simulated drought environment. The results showed that with increasing drought stress time net photosynthetic rate continuously decreased, and stomatal conductance and transpiration rate were similar to net photosynthetic rate for the seven seedling types. The intercellular CO_2_ concentration of *R. sinica* and *C. diplopetela* decreased initially and then increased, *F. microcarpa* increased first and then decreased, *A. nitidus* ssp. *lingnanensis* increased first, and then decreased followed by an increase, whereas other species decreased gradually. With increasing drought stress time, the stomatal limitation of *R. sinica* and *C. diplopetela* increased initially and then decreased, *F. microcarpa* decreased first and then increased, and *A. nitidus* ssp. *lingnanensis* decreased first, and then increased followed a decrease, whereas that of other species continuously increased. Principal component analysis indicated that drought resistance of the seedlings decreased in the order of *I. rotunda* > *F. microcarpa* > *A. nitidus* ssp. *Lingnanensis* > *P. sheareri* > *S. hainanense* > *C. diplopetela* > *R. sinica*.

**Keywords:** Drought stress; Seedling; Gas exchange parameter; Analysis of principal component

## 08 Changes of fluorescence parameters of greening seedlings of seven species under drought stress

### Jie Li, Zhuomin Wang, Li Xue

#### College of Forestry and Landscape Architecture, South China Agricultural University, Guangzhou 510642, China

##### **Correspondence:** Li Xue (forxue@scau.edu.cn)

We studied changes of the fluorescence indexes of the seedlings of *Syzygium hainanense, Phoebe sheareri, Radermachera sinica, Ilex rotunda, Ficus microcarpa, Carallia diplopetela* and *Artocarpus nitidus* ssp. *lingnanensis* through continuous drought. The results showed that with increasing drought time, the photochemical quantum yield of PSII in the light (Yield) of *S. hainanense, R. sinica, I. rotunda, F. microcarpa* and *C. diplopetela* gradually decreased, whereas that of *P. sheareri* and *A. nitidus* ssp. *lingnanensis* kept steady. With increasing drought time, the apparent electron transport rate (ETR) of *S. hainanense, R. sinica, I. rotunda, F. microcarpa* and *C. diplopetela* gradually decreased, whereas that of *P. sheareri* and *A. nitidus* ssp. *lingnanensis* kept steady. With increasing drought time, the non-photochemical quenching (NPQ) of *S. hainanense, P. sheareri, I. rotunda, F. microcarpa* and *A. nitidus* ssp. *lingnanensis* gradually increased, whereas that of *R. sinica* and *C. diplopetela* increased first and then decreased. Principal component analysis indicated that the order of drought resistance was *I. rotunda* > *F. microcarpa* > *A. nitidus* ssp. *lingnanensis* > *P. sheareri* > *S. hainanense* > *C. diplopetela* > *R. sinica.*

**Keywords:** Drought resistance; Fluorescence parameter; Principal component analysis; Seedlings

## 09 Mammalian sex hormone affects regeneration capacity and enzymes activity of Triticale L in vitro culture

### Ismail Bezirganoglu^1^, Pınar Uysal^2^

#### ^1^Deparment of Molecular Biology and Genetic, Erzurum Technical University, Erzurum 442, Turkey; ^2^Ministry of Food, Agriculture and Husbandry. Eastern Anatolia Agriculture Research Institute, Erzurum 442, Turkey

##### **Correspondence:** Ismail Bezirganoglu (ismail.bezirganoglu@erzurum.edu.tr)

This study is the first report that determines the effects of 17β-estradiol, estrone, progesterone and androsterone among the mammalian sex hormones on in vitro regeneration of Triticale mature embryos. A range of parameters which were 0 (control), 10^−4^, 10^−8^ and 10^−12^ m mol L^−1^ doses of 4 different mammalian sex hormones were investigated. It was clear that mature embryo interacted with the mammalian sex hormones. In mammalian sex hormones applications, estron group gave the best result in terms of explant percentage forming total shoots, followed by 10^−12^ m mol L^−1^ of progesterone. Moreover, effects of mammalian sex hormones on proline and activities of enzymes in vitro regenere plantlets were investigated. Proline and activities of enzyme significantly increased at all the concentrations tested compared to control group. The maximum enzyme activities were observed at the 10^−12^ m mol L^−1^ concentrations for all of three hormones in vitro culture.

## 10 Fractional Fourier entropy increases the recognition rate of fruit type detection

### Shuihua Wang^1^, Zhihai Lu^1^, Jiquan Yang^1^, Yudong Zhang^1,2^, John Liu^3^, Ling Wei^4^, Shufang Chen^5^, Preetha Phillips^6,7^, Zhengchao Dong^8^

#### ^1^Nanjing Normal University, Nanjing, Jiangsu 210023, China; ^2^Key laboratory of symbolic computation and knowledge engineering of ministry of education, Jilin University, Changchun, Jilin 130012, China; ^3^Michigan State University, East Lansing, MI 48823, USA; ^4^Shanghai Jiaotong University, Shanghai 200030, China; ^5^University of Electronic Science and Technology of China, Chengdu, Sichuan 611731, China; ^6^Shepherd University, Shepherdstown, WV 25443, USA; ^7^West Virginia University, Morgantown, WV, 26505, USA; ^8^New York State Psychiatric Institute, New York, NY 10032, USA

##### **Correspondence:** Yudong Zhang (zhangyudong@njnu.edu.cn)

**Aim:** To increase the recognition rate for the detection of fruit types, we carry out a research project.

**Materials:** The research team collected 1653 images that are composed of 18 fruit types as granny Smith apples, green grapes, tangerines, red grapes, Rome apples, blackberries, cantaloupes, Anjou pears, green Plantains, watermelons, Hass avocados, gold pineapples, passion fruits, bosc pears, black grapes, yellow bananas, blueberries, and strawberries.

**Method:** The four-step pre-processing was implemented to remove background and center the fruit. Afterwards, we used a new global feature – fractional Fourier entropy (FRFE) to extract features. The single-hidden layer feed-forward neural-network (SLFN) was utilized as the classifier. Back propagation algorithm was employed to train the network.

**Results:** Simulation results showed their proposed method achieved an overall accuracy of 88.99 % on the basis of a 5-fold cross validation.

**Conclusion:** The developed fruit-type detection system is promising and is now in preparation for practical use.

**Future Research:** Our next research direction is to introduce three-dimensional (3D) printing technique to offer 3D printed fruit, so the team can find more important and distinguishing features.

**Keywords:** Fruit classification; Detection; Recognition; Fractional Fourier entropy; Single-hidden layer feed-forward neural-network

**Acknowledgements**

Supported by Natural Science Foundation of Jiangsu Province (BK20150983).

## 11 Banana-peanut intercropping reduces *Fusarium* wilt disease in banana from enhancing soil bacterial microorganisms and leaf nutrition

### Hong Li, Xutong Wang, Fengliang Zhao, Guisheng Yang

#### Environment and Plant Protection Institute, Chinese Academy of Tropical Agricultural Science, Haikou, Hainan 571101, China

##### **Correspondence:** Hong Li (hongliss@hotmail.ca)

Banana (*Musa acuminata) Fusarium* wilt caused by the fungal pathogen *Fusarium oxysporum* is a soil-borne, destructive disease worldwide. Our objectives were to examine whether banana intercropping with N-fixing peanut crops could enhance soil microorganisms and improve plant nutrition leading to banana resistance to *Fusarium* infection. The experiments were conducted in 6 separate fields (0.5-ha each) in Hainan Island, southern China during 2015-2016. Banana seedlings were transplanted in 2 × 2 m spacing and peanuts were seeded in 0.25 × 0.5 m spacing in each banana row. Each field 0.1-ha areas were kept as non-intercropping reference. After harvest all peanut crop residues were incorporated into banana rooting zones. By fruit formation stage, intercropped banana plants were healthy with only 2 % of the plants showing wilt disease symptoms against 19 % for the non-intercropped plants. Soil organic carbon increased from 18.4 mg/g to 22.3 mg/g. The cultured bacteria and actinomycetes significantly increased by 23 % and 45 % respectively but there was a decrease in 6.9 % for cultured fungus. Banana leaf N content was significantly higher (4.1 ± 0.6 %) in the intercropping plots compared to the reference plants (3.5 ± 0.7 %). Together enhanced soil microorganisms and improved leaf N nutrition could promote plant resistance to *Fusarium* infection. Adoption of intercropping with legume crops would be practical for reducing *Fusarium* disease in banana production.

## 12 Manganese stress impairs stem ureide nitrogen fixation in yardlong-bean plants in the acidic environment

### Hong Li (hongliss@hotmail.ca)

#### Environment and Plant Protection Institute, Chinese Academy of Tropical Agricultural Science, Haikou, Hainan 571101, China

Manganese (Mn), an essential micronutrient for plant growth, involves in plant photosynthetic and metabolic processes. Yet soil Mn stress, organic-C and rhizobial inoculant interactions on legume symbiotic N fixation are not well understood. Our objectives were to examine crop tolerance to Mn stress and to determine the relationships between Mn uptake, root nodulation, stem ureide, amino-N and nitrate holding in yardlong-beans (*Vigna unguiculata* subsp*. sesquipedalis*), one of the major tropical fresh vegetables. The field studies were conducted in Hainan, southern China during 2012-2014. The experimental treatments consisted of three levels of organic C (OC, 0, 4 and 8 Mg/ha estimated from chicken compost) and four levels of rhizobial inoculants (RI, 0, 6, 12 and 18 g/kg), arranged in a split-plot design with 4 replicates. The results showed that yardlong-bean plants responded strongly to both OC and RI treatments in all measured physiological and nutritional aspects (*P* < 0.05). Leaf Mn holding varied between 496 ± 89 μg/g. Plant Mn in excess (>360 μg/g) could induce plant stress to impair leaf chlorophyll, stomatal conductance, carbonxylation, stem allantoin, amino acids, nitrate concentrations and plant N derived from N fixation in legume crops. The OC and IR antagonistic interactions could increase crop tolerance to Mn stress from promoting stem ureide development and amino acid holding.

**Keywords:** Manganese stress; Photosynthesis; Stem ureide; Nitrogen fixation; Yardlong-beans

## 13 A new pest control method for *Rhytidodera bowringii* Larvae

### Haijie Huang^1^, Li Zhao^2^, Weijian Huang^1^, Jinhui Wang^1^, Zhongrun Zhang^1^

#### ^1^Tropical Crops Genetic Resources Institute, CATAS, Danzhou, 571737, China; ^2^College of Applied Science and Technology, Hainan University, Danzhou, 571737, China

##### **Correspondence:** Haijie Huang (huanghaijie1979@163.com)

*Rhytidodera bowringii* larvae are a tropical fruit pest. They burrow into trunks of mango, cashew, and other tree, which damages portions of the plants. The damage caused by the larvae can have significant negative effects on fruit quality and production. Currently, common pest control methods for *R. bowringii* include hunting, chemical, and biological approaches. All these methods are all fairly inefficient. Thus, it is important to develop a efficient and environment friendly control method for *R. bowringii* larvae. In this study, infected cashew trunks were used as research subjects. Holes chewed by *R. bowringii* larvae were blocked with cotton balls dipped in different concentrations of deltamethrin, and then wrapped tightly with polyethylene films. Five days later, the infected trunks were cut off to determine the status of the pests. The results showed that blocking holes with cotton balls dipped in 2.5 % (V/V) deltamethrin and wrapping the branch with polyethylene film can kill all of the larvae and eggs. This method is labor intensive but environmental friendly, and can be used on small scale to control *R. bowringii*.

**Acknowledgements**

This research was supported by National Nonprofit Institute Research Grant of CATAS-TCGRI (1630032015033) and The Ministry of Agriculture Tropical Crop Germplasm Resources Protection (16RZZY- 101).

## 14 Research on the seed-like Fruits of Subg. Sclarea of *Salvia* of Labiatae in China

### Xiaojuan Li^1^, Ning Xu^1^, Guofu Zhou^1^, Ming Wan^1^, Qi Lin^5^, Fanyun Meng^1,2^, Jianxiu Li^3,4^

#### ^1^Beijing Area Major Laboratory of Protection and Utilization of Traditional Chinese Medicine, Beijing Normal University, Beijing 100875, China; ^2^State Key Laboratory Breeding Base of Dao-di Herbs, National Resource Center for Chinese Materia Medica, China Academy of Chinese Medical Sciences, Beijing, 100700, China; ^3^Shandong University of Traditional Chinense Medicine, Jinan, 250014, China; ^4^Shandong Hongjitang Museum, Jinan, 250103, China; ^5^Institute of Botany, the Chinese Academy of Sciences, Beijing, 100093, China

##### **Correspondence:** Fanyun Meng (mfy@bnu.edu.cn); Jianxiu Li (jianxiu_li@163.com)

Four sect. of seed-like Fruits (including 14 species) of Subg. Sclarea of *Salvia* of Labiatae in China were systematically observed and researched by using both biological anatomical lens and scanning electron microscope (98 photographs were attached). Their shapes were ovoid, elliptical and fusiform and so on; the sizes were large, medium and small; the surface ultrastructures of seed-like Fruits were ranged from plain, stripes, reticulate to convex meshes and the like. The relationships among them and the position of evolution and the relationships among three Ser. of Sect. Drymosphace were discussed. According to the surface ornamentation types analysis of seed-like Fruits, their evolution was from plain, stripes, reticulate, convex meshes to reticulate and convex meshes, and both reticulate and convex meshes appeared on dorsal and ventral surfaces of seed-like Fruits of one species at the same time. The plain was an original type; the stripes, reticulate and convex meshes were intermediate types; and both reticulate and convex meshes appeared on seed-like Fruits of one species was regarded as an evolution type. This was a rare phenomenon in other Family and Genus of angiosperms, which was worthy of an in-depth study. According to types of the ornamentation characteristics, they had intra-specific stabilities, but significant inter-specific difference in both the macro morphology features and the ultrastructure ornamentation, which provided evidences for classification identification, discussion on their genetic relationships, introduction and cultivation and genetics and breeding.

**Keywords:** Labiatae; *Salvia* ; Subg. Sclarea; Seed-like Fruits; Ultrastructures; SEM

## 15 Three pulling resistance models of pioneer plant in landslide area

### Yichang Chen^1^, Koayung Yu^2^, Chunpin Chang^3^

#### ^1^National Chung Hsing University, Taichung, Taiwan, 402, R.O.C.; ^2^Ling Tung University, Taichung, Taiwan, 408, R.O.C.; ^3^Chung Chou University of Science and Technology, Changhua, Taiwan, 510, R.O.C.

##### **Correspondence:** Chunpin Chang (plus871014@gmail.com)

Vegetation in landslide area is difficult because of barren, water stress, coarse-textures soils, and steep slopes etc. In this study we need to construct the criteria of slopeland protection for the selected vegetation materials in considering their characteristics and the soil solidities for the root system. With the selected three pioneer plants, India-charcoal Trema (Trema orientalis Blume), Formosan alder (Alnus formosana Makino) and Roxburgh sumac (Rhus javonica L. var. roxburghiana), the destructive pulling resistance (Pr) models, with the weight of plant above the ground (Wu), the weight of root-soil (Wd) or the diameter of the tree just above the ground (D), The models were Pr = 0.469Wu, Pr = 2.9561D + 0.0234Wd and Pr = 1.0184D + 0.0802Wu in proper order. And the non-destructive resistance models, with the D, were derived with multi-variable regression analysis, respectively. The models were Pr = 2.882D, Pr = 3.1552D and Pr = 2.2433D in proper order. The models gave higher statistical regression coefficients when they were compared with the results of relative researches. The significant level factors to influence the plant pulling resistance capacity are climate and soil properties. Finally the relevant conclusions were obtained.

**Keywords:** Landslide area; Pioneer plant; Pulling resistance; Root system

## 16 The comparison of physiological and biochemical mechanisms of *Reaumuria soongorica* and *Salsola passerine* in different growth pattern

### Zijuan Zhou, Peixi Su, Rui Shi, Tingting Xie

#### Key Laboratory of Land Surface Process and Climate Change in Cold and Arid Regions, Cold and Arid Regions Environmental and Engineering Research Institute, Chinese Academy of Sciences, Lanzhou 730000, China

##### **Correspondence:** Zijuan Zhou (zhouzzj@lzb.ac.cn)

The C_3_ plant *Reaumuria soongorica* and C_4_ plant *Salsola passerina* are super-xerophytes that co-exist in desert ecosystems of China. The species show a unique growth patterns in growing either in individual (isolated growth) or in associated communities (associated growth) that may favour adaptation to the extreme conditions. In this study, leaf water status, chlorophyll content, antioxidant enzyme activities, and contents of solutes contributing to osmotic adjustment were investigated in plants growing in individual and mixed communities. The objective was to identify physiological and biochemical differences in the two growth patterns. In associated communities the chlorophyll contents of *R. soongorica* and *S. passerina* were decreased, whereas superoxide dismutase and peroxidase activities were enhanced. The contents of osmotic adjustment substances differed between the two species, with *R. soongorica* showing higher soluble sugar and proline contents than those of *S. passerina*. Furthermore, the soluble sugar and soluble protein contents were elevated in associated growth. It is concluded that growth in associated communities may improve antioxidant scavenging and the osmotic adjustment capacity of the two species, which will alleviate the adverse effects of the desert environment, and that association in mixed communities is more conducive to plant water uptake and enhances drought tolerance.

## 17 Resources use efficiency of the cosmopolitan plant *Potentilla anserina* L. in different alpine habitats in China

### Rui Shi, Peixi Su, Zijuan Zhou

#### Key Laboratory of Land Surface Process and Climate Change in Cold and Arid Regions, Cold and Arid Regions Environmental and Engineering Research Institute, Chinese Academy of Sciences, Lanzhou 730000, China

##### **Correspondence:** Rui Shi (18634936969@163.com)

*Potentilla anserina* L. is a psychrophyte of high nutritional and medicinal value that is widely distributed in the alpine region of Southwest China. In this study, three communities—swamp, wet meadow and dry meadow—were selected at the same latitude to represent the gradient in groundwater level, surface water level and soil moisture in the Zoige grassland on the Eastern Qinghai-Tibetan Plateau. The abundance and reproductive capacity of *Potentilla anserina* in the communities were investigated, and the CO_2_ assimilation rate of *P. anserina* was measured to estimate water use efficiency (WUE) and light use efficiency (LUE). The net photosynthetic rate (*P*_n_), WUE and LUE of *P. anserina* differed significantly among habitats (*P* < 0.05). The *P*_n_, LUE and WUE were lowest in swamp, whereas maximum *P*_n_ and LUE were observed in wet meadow and maximum WUE was recorded in dry meadow. In the regressive succession from swamp to wet meadow to dry meadow, *P. anserina* abundance increased and reproductive capacity was enhanced; the light compensation point decreased, the light saturation point increased, and the carbon assimilation time was extended. It is concluded that *P. anserina* shows characteristics suited for alpine meadow regressive succession and is an indicator species for alpine meadow degradation.

**Acknowledgements**

Supported by the National Key Basic Research Program of China (Grand No. 2013CB956604).

## 18 Cloning of PPDK gene from *Red Amaran*and transformation of *Alfalfa*

### Xuelan Liu, Yan Zhang, Xiangfa Wei

#### Poultry Research Institute, Shandong Academy of Agricultural Sciences, Jinan 250023, P. R. China

##### **Correspondence:** Xuelan Liu (jqsliuxl@163.com)

**Background:** Cloning valuable genes can provide useful objectives for plant engineering. Pyruvate orthophosphate dikinase (PPDK), the limited enzyme in C_4_ photosynthesis and crassulacean acid metabolism (CAM).Some research indicated that over-express the gene encoding PPDK could improve the photosynthesis rate and the production. In this study, the C_4_ type-PPDK gene in *Red amaran* was cloned and transformed into *alfalfa*.

**Results:** The gene encoding C_4_ type-PPDK in *Red amaran* has been cloned; it showed that the PPDK cDNA was 3.4 kb, including an ORF of 2871 bp, which encoding of 957 AA of 107kD. By comparing, there is a highest similarity with *wheatgrass* of 84 %. Plant expression vector containing the full length of C_4_-type PPDK gene was constructed and transgenic *alfalfa* plants were obtained. A sequence about 600 bp in the upstream of the C_4_ type-PPDK cDNA was cloned, it contains TATA-box, CAAT-box, GT1-motif and sp1, some was transformed into tobacco, and the result showed that there was a transient expression in it.

**Conclusion:** PPDK gene of type C_4_ plants will improve the photosynthetic efficiency of C_3_ plants.

**Keywords:** *Red amaranth*, PPDK, *Alfalfa*

## 19 Variation and cluster analysis of morphological characters and nutrient content of *Chucrasia tabularis* seed

### Chong Wu, Yanlei Yin, Lijuan Feng, Xuemei Yang, Fei Wang

#### Shandong Institute of Pomology, Taian 271000, Shandong, China

##### **Correspondence:** Chong Wu (wuchongge@163.com)

This study was conducted to investigate the variation in seed traits and nutrient contents among 26 provenances of *Chukrasia tabularis* from 8 countries in Asia and Australia. There were highly significant differences among provenances in seed length, width, thickness, seed length-width ratio, 100-seed weight, contents of soluble protein, soluble sugar and starch in seed. Although provenances showed mixed performances across the seed parameters measured, seeds from Australia and Malaysia were generally larger and heavier, and had higher protein, sugar and starch contents than those from other countries. Significant correlations between seed traits and environmental attributes were observed. Nutrient contents were correlated negatively with altitude, suggesting that provenances from lower altitude had higher nutrient contents, and vice versa. Principal component analysis revealed that seed length, width and weight, and soluble protein, soluble sugar and starch contents were useful characteristics in explaining the variation pattern among provenances. This study indicates marked variation in seed size parameters and nutrient content among *C. tabularis* provenances and the influence of geographic location and climate on the variation.

**Keywords:** *Chukrasia tabularis*; Provenance; Seed character; Nutrient; Cluster analysis

**Acknowledgements**

Supported by a project grant from Shandong Academy of Agricultural Youth Fund (2016YQN31), Shandong Institute of Pomology Fund.

## 20 Effect of the planting density of the areca nut on the growth of intercropped Vanilla

### Hua Wang^1,2^, Huifa Zhuang^1^, Zihui Zhu^1^, Hui Wang^1^

#### ^1^Institute of Spice and Beverage Research, CATAS, Wanning 571533, China; ^2^Tropical Crops Genetic Resources Institute, CATAS, Danzhou 571737, China

##### **Correspondence:** Hui Wang (wanghui_gz@163.com)

Vanilla (*Vanilla planifolia* Jacks) is a tropical spice crop that is suitable for intercropping with the areca nut (*Areca catechu* L.). A clear understanding of the effect of the planting density of the areca nut on the growth of intercropped vanilla is important for the rational distribution of the intercropping systems. In this study, three areca nut planting densities with vanilla intercropping were set up: 2.0 × 2.5 m, 2.0 × 3.0 m, and 2.5 × 2.5 m, using an artificially shaded vanilla monoculture as the control. The biomass, the photosynthetic characteristics of the leaves, and the chlorophyll fluorescence characteristics of the vanilla were analyzed. It was found that the areca nut intercropping could significantly increase the stem growth and photosynthetic rate of vanilla. The treatment of an areca nut planting density of 2.0 × 2.5 m produced the maximum vanilla stem biomass as well as the best photosynthetic characteristics of the leaves, including the potential photochemical efficiency, the apparent electron transport rate, the maximum photochemical efficiency, and the actual photochemical efficiency of photosystem II. Therefore, an areca nut planting density of 2.0 × 2.5 m is optimal for vanilla intercropping.

